# Effect of feeding goat meat containing low cholesterol and rich omega-6 fatty acid on blood lipid status of white rat (*Rattus norvegicus*)

**DOI:** 10.14202/vetworld.2021.1966-1970

**Published:** 2021-07-29

**Authors:** Widiyanto Widiyanto, Mulyono Mulyono, Sutrisno Sutrisno, Eko Pangestu, Marry Christiyanto, Surahmanto Surahmanto, Vitus Dwi Yunianto, Bambang Waluyo Hadi Eko Prasetiyono

**Affiliations:** Department of Animal Science, Faculty of Animal and Agricultural Sciences, Diponegoro University, Tembalang Undip Campus, Semarang, Indonesia

**Keywords:** atherogenic index, blood lipid status, cholesterol, goat meat, omega-6 fatty acids, white rat

## Abstract

**Background and Aim:**

Healthy goat meat is an essential aspect in increasing consumer acceptance for this livestock product. The research aimed to examine the effect of goat meat containing low cholesterol and rich omega-6 fatty acid on the performance and blood lipid status of white rats (*Rattus norvegicus*).

**Materials and Methods:**

Thirty 2-month-old male white rats (*R. norvegicus*) weighing 195-230 g were randomly divided into three groups, with each group consisting of 10 rats. Group I was treated with a control feed (T0; BR I concentrate). Group II (T1) was treated with a mixed feed containing 50% control feed and 50% goat meat. Group III (T2) was treated with a mixed feed comprising 50% control feed and 50% goat meat with low cholesterol and rich omega-6 fatty acids. Each treatment was given *ad libitum* for 30 days. The variables measured were dry matter and organic matter consumption, daily body weight gain, feed conversion, triglyceride levels, total cholesterol, high-density lipoprotein (HDL) and low-density lipoprotein (LDL) cholesterol, and atherogenic index (AI). The data were analyzed statistically using analysis of variance in a completely randomized design.

**Results:**

The total, HDL, and LDL cholesterol levels at T0, T1, and T2 were as follows: 99.97, 35.97, and 50.43 mg/dL (total cholesterol); 108.35, 33.92, and 58.17 mg/dL (HDL cholesterol); and 101.43, 38.09, and 48.65 mg/dL (LDL cholesterol). The highest HDL and the lowest LDL cholesterol levels (p<0.05) were observed in the T2 treatment group, which had the lowest AI (1.69 vs. 1.77 and 2.19).

**Conclusion:**

The consumption of goat with low cholesterol and rich omega-6 fatty acids reduces the total cholesterol and LDL cholesterol, raises the HDL cholesterol levels, and decreases the AI.

## Introduction

The supplementation of protected polyunsaturated fatty acid (PUFA) sources is a technology that can increase the productivity of ruminants through increasing the metabolic rate and, in turn, increasing the livestock product biosynthesis, that is, the ruminant meat. PUFA protection will allow these nutrients to be absorbed in the intestine without the biohydrogenation process of the rumen, thus improving the livestock lipid status by increasing the high-density lipoprotein (HDL), decreasing low-density lipoprotein (LDL), and controlling blood cholesterol [1]. PUFA supplementation also allows compounds to be deposited in ruminant tissue, and as a result, the ruminant meat contains low cholesterol levels but is rich in the essential PUFAs. The consumption of such ruminant products will improve the health of consumers because the mechanism for enhancing the lipid status can also occur in consumers of these livestock products [1].

The wide application of PUFA source supplementation technology in the field is expected to increase the productivity of small ruminants while at the same time increasing meat preference. Thus, such technology can contribute significantly to efforts aimed at increasing small ruminant meat consumption to support the diversification of meat consumption. To ensure the wide and successful application of this technology, scholars proved that the consumption of livestock products (in this case, meat) of small ruminants, which are low in cholesterol and rich in omega-6 fatty acids, can nourish the consumers to a healthy status, which is reflected in their blood lipid status. The proof was based on the results of the previous studies, which have developed the technology to produce small ruminant meat (in this case, *Kacang* goats) that is low in cholesterol and rich in omega-6 fatty acids through the supplementation of protected PUFAs in combination with methionine hydroxy analogs (MHAs) (Table-1).

**Table-1:** Nutrient composition of treatment ration component.

Nutrient	BR I	KGM	KGMLC
Crude protein (%)^a^	20.73	72.95	81.46
Lipid (%)a	6.93	19.18	10.04
Crude fiber (%)^a^	3.09	-	-
Gross energy (cal/g)*^a^*	3871	6291	5027
Cholesterol (mg/100g)^b^	-	89.37	67.18
Linoleic acid (%)^c^	-	5.39	14.93
Iodine number of fat		5.17	11.31

KGM=*Kacang* goat meat, KGMLC=KGM with low cholesterol and rich omega-6 fatty acid,

a =Dry matter (DM) basis,

b =mg cholesterol per 100 g fresh meat,

C =Relative proportion of total fatty acids in the meat

This study aimed to prove that the consumption of goat meat that has low cholesterol and is rich in omega-6 fatty acids is healthy for consumers. The results of the study on laboratory animals became an indicator of the positive effect of such practice, which was reflected in the consumer lipid status, including total cholesterol, HDL cholesterol, LDL cholesterol, and atherogenic index (AI) [2]. This study used white rats (*Rattus norvegicus*) because they are physiologically and metabolically similar to humans [3]. The dissemination of the results of this study is expected to increase consumer preferences and the demand for small ruminant products, in this case, goat meat, which, in turn, can promote the increased productivity of livestock commodities.

## Materials and Methods

### Ethical approval

All the procedures for experimental animal handling have been approved by the welfare and use committee of laboratory animal with protocol number: 074/B.1-KEPK/SA-FKG/XI/2018.

### Study period and location

The study was conducted from July to October 2019 at the Feed and Nutrition Laboratory, Faculty of Animal and Agricultural Sciences, Diponegoro University in Semarang, Indonesia.

### Animals and ration

As many as 30 heads of male white rats (*R. norvegicus*; 2 months old with body weights of 195-230 g) were used as the experimental material. The laboratory animals were obtained from the Biology Laboratory, Semarang State University. The feed given in the form of BR I feed was obtained from “Bamboo” Poultry shop, whereas *Kacang* goat meat (KGM) and KGM with low cholesterol and rich omega-6 fatty acids (KGMLC) were the results of raising *Kacang* goats in the nutrition and feed science laboratory, Faculty of Animal Husbandry and Agriculture at Diponegoro University, Semarang. The production of KGMLC was carried out by rearing the *Kacang* goat treated by protected kapok seed oil supplementation in combination with MHA, in accordance with the previous research procedures. The meat used in the treatment was a longissimus dorsi muscle. Table-1 shows the nutrient composition of BR I, KGM, and KGMLC feeds.

### Experimental design and blood sampling

The research animals were randomly divided into three groups, with each group consisting of 10 heads as replications. Group 1 was fed with BR I (control), Group II received a mixture of BR I and KGM feed (50%:50%), whereas Group III received a feed mixture of BR I and KGMLC feed with 50%:50% proportions. Tables-2 and 3 list the nutrient composition of the treatment ration.

**Table-2:** Composition of treatment ration.

Item	T0	T1	T2
BR I feed (%)	100	50	50
KGM (%)	0	50	-
KGMLC (%)	0	0	50

T0=The ration consisting of 100% BR I feed, T1=The ration consisting of 50% BR I feed and 50% KGM, T2=The ration consisting of 50% BR I feed and 50% KGMLC. KGM=*Kacang* goat meat, KGMLC=KGM with low cholesterol and rich omega-6 fatty acid

**Table-3:** Nutrient composition of treatment ration (DM basis).

Nutrient	T0	T1	T2
Crude protein (%)	20.73	46.82	51.09
Lipid (%)	6.93	13.05	8.48
Crude fiber (%)	3.09	1.54	1.54
Gross energy (cal/g)	3871	5081	4449

T0=The ration consisting of 100% BR I feed, T1=The ration consisting of 50% BR I feed and 50% KGM, T2=The ration consisting of 50% BR I feed and 50% KGMLC. KGM=*Kacang* goat meat, KGMLC=KGM with low cholesterol and rich omega-6 fatty acid

The animals were sedated with ketamine HCl (1 mg/100 g body weight) at the beginning and end of the study for weighing and blood collection. Blood was obtained by amputation of the tail and stored in a small bottle (vial) and then centrifuged at 3000 rpm for 5 min. The supernatant was collected by a pipette and stored at −4°C until analysis. The feed was weighed before and after feeding every day to calculate the daily average feed consumption per head.

Measurements of triglyceride (TG), total cholesterol, and HDL and LDL cholesterol levels of the blood serum were carried out using the colorimetric enzymatic principle [4]. AI was calculated by the formula AI=(total-HDL cholesterol)/HDL cholesterol [5]. The data of body weight gain (BWG), feed consumption, feed conversion, TG levels, total cholesterol, HDL and LDL cholesterol, and IA were analyzed statistically in a completely randomized design, in accordance with the work of Steel *et al*. [6].

## Results and Discussion

Studies about the effect of consumption of KGMLC included the aspects of consumer performance and blood lipid status. The analysis of the results of this study was conducted by comparing the effects of consumption of KGMLC ration to the consumption of KGM ration and consumption of BR I feed as control using male white rats (*R. norvegicus*) as experimental animals.

### Performance of the experimental animals

The influence of feed treatment on the performance of experimental animals was reflected in several variables, including the DM consumption of feed, daily BWG (DBWG), and feed conversion (Table-4). The DM consumption in the KGM treatment group was higher (p<0.05) than that in the control group (17 vs. 13 g). This result was thought to be due to the high palatability of KGM than the control group (C).

**Table-4:** Feed consumption, DBWG, and feed conversion.

Treatment ration	Daily feed consumption (g)	Daily body weight gain (g)	Feed conversion
T0 (C)	13.39^b^	1.27^c^	10.92^a^
T1 (KGM)	17.38^a^	1.53^b^	11.50^a^
T2 (KGMLC)	12.92^b^	1.75^a^	7.55^b^

T0=The ration consisting of 100% BR I feed, T1=The ration consisting of 50% BR I feed goat and 50% KGM, T2=The ration consisting of 50% BR I feed and 50% meat that is low in cholesterol and rich in omega-6 fatty acid (KGMLC). a, b, and c=Different superscripts in the same row show significant differences (p<0.05). KGM=*Kacang* goat meat, KGMLC=KGM with low cholesterol and rich omega-6 fatty acid

The white rats that were used as experimental animals were about 2 months old, still growing, and in puberty. The secretion of growth hormone and testosterone is very active in this physiological phase. Androgen hormones play a role in bone growth and maturation, collagen synthesis, and bone mineralization [7]. Increasing the protein intake supports the biosynthetic process, which, in turn, increases the energy requirements and promotes the increased consumption of feed DM [8]. The resulting increase in DM consumption, which increased protein and energy supply, is also a synergistic anabolic role of growth hormone and testosterone. This condition resulted in the higher DBWG in the T1 treatment group (p<0.05) compared with the control group (T0), that is, 1.27 versus 1.53 g.

The treatment group that received KGMLC exhibited a low feed consumption although their BWG was higher than that of the other treatment groups (p<0.05). The higher BWG in the T2 treatment group compared with T0 was caused by the higher protein and energy intake, thus implying the higher protein and energy levels in the T2 ration compared with the T0 treatment group. An interesting phenomenon observed in the lower feed consumption of the T2 treatment group (p<0.05) than T1 (12 vs. 17 g) was its higher DBWG (p<0.05) than T1 (1.75 vs. 1.53 g) although the levels of crude protein and energy of T2 ration showed no considerable difference from those of T1 (Table-3). The T1 and T2 treatment groups exhibited no considerable difference in their protein and energy levels (46.82% and 5081 cal/g for T1; 51.09% and 4449 cal/g for T2). The protein and fat requirements for growing the white rats based on Nutrient Research Council [9] were 15% and 5%, respectively. After reaching the maximum growth rate, the rats in the T1 treatment group used the excess energy and protein for lipid deposition in adipose tissues. The T2 treatment group had a higher tissue protein biosynthesis but a lower lipid deposition than the T1 treatment group. Thus, the former presented a higher DBWG than former, although the feed consumption in the T1 treatment group was higher than that in the T2 treatment group.

The relatively high proportion of linoleic acid in KGMLC, which allowed the intake of these essential fatty acids at T2, was higher than that of the other treatment groups. Omega-6 fatty acids, given their position as structural components of cell membranes, have a very important biological role in metabolism. This role is expressed through their function to maintain the membrane integrity and as a second messenger that facilitates the entry of biosynthetic precursors and the increase in intracellular metabolism [10]. Linoleic acid allowed the increase in tissue protein biosynthesis in the T2 treatment group, whose value was higher than that of the other treatment groups, thus resulting in their higher BWG (p<0.05) compared with the other treatment groups.

Lipid deposition in the T2 treatment group was lower than that in the T1 treatment group due to the increased linoleic acid intake of the former. According to Airaodion *et al*. [11], PUFAs with cis configuration (including linoleic acid) inhibit the biosynthesis of long-chain fatty acids in adipose tissues. This phenomenon occurs because PUFAs inhibit RNA transcription, which regulates the production of fatty acid synthetase enzymes, thus inhibiting the *de novo* fatty acid biosynthesis in adipose tissues. The inhibition of *de novo* fatty acid biosynthesis led to a response to the high-energy consumption in the form of a decreased DM consumption in the T2 treatment group, which resulted in a lower value compared with those of T1 and T0.

The lowest consumption of DM with the highest DBWG in the T2 treatment group produced the lowest feed conversion compared with the T1 (KGM) and T0 (BR I) treatment groups. DM consumption in the T1 treatment group was higher than that in T0, resulting in a feed conversion that was not significantly different from that in T0 because the DBWG of experimental animals at T1 was higher than that at T0.

### Blood lipid status of experimental animals

The blood lipid status of the experimental subjects was analyzed based on TG, total cholesterol, and HDL and LDL cholesterol levels. The tendency of feed to cause atherosclerosis was also assessed based on the AI.

The blood-serum TG is mainly derived from the absorption following its synthesis in intestinal cells, which are packaged in chylomicron and synthesized in the liver and are then transported in the form of very LDLs (VLDLs) [12]. Blood-serum TG level in the T1 treatment group (83 mg/dL) was higher (p<0.05) than those in the T0 and T2 treatment groups (59 and 62 mg/dL, respectively; Table-5). This result was caused by the higher lipid content and feed consumption in the T1 treatment group than in T0. Thus, the fatty acids and glycerol available for esterification in the T1 treatment group were also higher than those of the T0 treatment group.

**Table-5:** Blood-serum lipid status.

Variable	T0	T1	T2
Triglyceride (mg/dL)	59^b^	83^a^	62^b^
Total cholesterol (mg/dL)	99.97^b^	108.35^a^	101.43^b^
High-density lipoprotein cholesterol (mg/dL)	35.97^b^	33.92^c^	38.09^a^
Low-density lipoprotein cholesterol (mg/dL)	50.43^b^	58.17^a^	48.65^b^
Atherogenic index	1.77^b^	2.19^a^	1.69^b^

T0=The ration consists of 100% BR I feed, T1=The ration consists of 50% BR I feed goat and 50% KGM, T2=The ration consists of 50% BR I feed and 50% KGMLC. a, b, and c=Different superscripts in the same row show a significant difference (p<0.05). KGM=*Kacang* goat meat, KGMLC=KGM with low cholesterol and rich omega-6 fatty acid

Lipid consumption in the T2 treatment group was higher than that in T0, although the consumption of DM was lower, because the lipid content in T2 rations was remarkably higher. However, the blood TG levels in T2 showed no significant difference from T0. One of the causes of this phenomenon is the inhibition of *de novo* fatty acid biosynthesis in the liver for esterification to TG by PUFAs from KGMLC. Another cause was the low absorption of lipid in TG form in the intestine. This event can occur because in the process of transport by PUFAs from the small intestine, TG is esterified in the form of ester cholesteryl and phospholipid [12].

Blood cholesterol is a free cholesterol and ester cholesteryl and is a component of lipoproteins for lipid transport [11]. Blood plasma cholesterol is mostly synthesized in the small intestine mucosal cells as a component of chylomicron to transport absorbed lipids, mainly TG. Blood-serum cholesterol is also a component of VLDL, which is mostly synthesized in the liver that enables the transport of TG from the liver to various extrahepatic tissues [13]. The total cholesterol levels in the T0, T1, and T2 treatment groups were 99.97, 108.35, and 101.43 mg/dL, respectively. The T1 treatment group exhibited the highest total cholesterol level in blood serum (p<0.05) followed successively T2 and T0 treatment groups. This phenomenon is in line with the level of fat consumption, which reached 0.90, 2.22, and 1.02 g/head/day in the T0, T1, and T2 treatment groups, respectively. The pattern of blood-serum cholesterol levels, which is related to fat absorption, was also reflected in the blood TG levels, which exhibited a similar pattern to blood cholesterol levels (Table-5).

The iodine number of KGM was lower than that of KGMLC (5.17 vs. 11.31) (Table-1), which reflects that the unsaturation degree of KGM fatty acids was notably higher than that of KGMLC. According to Shrestha *et al*. [14], unsaturated fatty acids can stimulate cholesterol excretion through the intestine and stimulate cholesterol oxidation into bile acids. Cholesteryl esters from unsaturated fatty acids are metabolized faster than cholesteryl esters from saturated fatty acids in the liver and other tissues, thus speeding up their excretion. This mechanism was thought to play a role in reducing the blood-serum total cholesterol levels in the T2 treatment group, which were lower than those of T1 but showed no significant difference from those of the control (T0).

Table-1 shows that KGMLC had a considerably higher relative proportion of linoleic acid than KGM (14.93% vs. 5.39%). According to Zhou *et al*. [15], the absorbed linoleic acid will be preferentially esterified in lysolecithin to form phosphatidylcholine or lecithin. Lecithin is the main phospholipid in HDL. The high intake of linoleic acid thus increases the fraction of ester cholesteryl and HDL phospholipids in the blood serum. Esterification of cholesterol is reflected in the high levels of HDL cholesterol in the blood serum. This phenomenon was observed in the T2 treatment group. Thus, the blood-serum HDL level in the T2 treatment group was higher than those of T1 and T0 treatment groups. The lowest HDL cholesterol level was recorded in the T1 treatment group, which consumed a lower KGMLC than the T2 treatment group.

The blood-serum LDL cholesterol levels in the T0, T1, and T2 treatment groups were 50.43, 58.17, and 48.65 mg/dL, respectively (Table-5). The lower LDL levels in the T2 and T0 treatment groups compared with the T1 treatment group (p<0.05) were caused by the lower fat consumption in the former compared with the latter. The absorbed fat enters the circulatory system in the form of chylomicron to supply fatty acids to various tissues, whereas the rest is carried to the liver. TGs and cholesterol that exceed the liver requirements are transported into the blood in the form of VLDL, which is then hydrolyzed by lipoprotein lipase on the capillary wall surface, leaving intermediate density lipoprotein and then LDL [16].

Another factor associated with a low LDL cholesterol level is the increase in HDL cholesterol level. Mireille *et al*. [17] stated that HDL facilitates the capture of blood cholesterol from other lipoproteins to be supplied to cells of various tissues and facilitates the capture and/or transport of cholesterol from extrahepatic tissues to the liver. This phenomenon resulted in lower LDL cholesterol levels in the T2 treatment group (p<0.05) compared with the T1 and T0 (control) treatment groups.

Table-5 indicates that the AI in the T1 treatment group (2.19) was higher (p<0.05) than that of the T2 and T0 treatment groups (1.69 and 1.77, respectively). This phenomenon was caused by the higher total cholesterol and lower HDL cholesterol levels in the T1 treatment group compared with the other treatment groups. The AI is one of the accurate measures to predict the risk of atherosclerosis [5,18,19].

## Conclusion

Based on the results of the study and their analysis, the consumption of up to 50% rations KGMLC by white rats (*R. norvegicus*) resulted in a better performance than the control and experimental animals that consumed KGM in the same proportions. The consumption of KGMLC up to 50% level of the diet improves the blood lipid status, which is reflected in the increased HDL cholesterol and decreased TGs, total cholesterol, LDL cholesterol levels, and AI.

## Authors’ Contributions

WW, BWHEP, MM, SS1, EP, MC, SS2, and VDY: Designed the study, wrote the manuscript, and participated in conducting the experiment. WW and BWHEP: Performed the *in vivo* experiment and biochemical investigations and collected the samples. MM and SRH: Processed and analyzed the data. All authors read and approved the final manuscript.
